# Long term effects of a health promotion intervention in low socioeconomic Arab- Israeli kindergartens

**DOI:** 10.1186/1471-2431-13-45

**Published:** 2013-04-01

**Authors:** Dan Nemet, Dganit Geva, Michal Pantanowitz, Narmen Igbaria, Yoav Meckel, Alon Eliakim

**Affiliations:** 1 Child Health and Sports Center, Pediatric Department, Meir Medical Center, Kfar-Saba, Sackler School of Medicine, Tel-Aviv University, 59 Tchernichovski Street, Kfar-Saba, 44281, Israel; 2 Zinman College of Physical Education and Sport Sciences at the Wingate Institute, Netanya, Israel

**Keywords:** Obesity, Preschool, Exercise, Nutrition, Intervention, Education, Long-term

## Abstract

**Background:**

Obesity is the most common chronic pediatric disease in westernized, especially low socioeconomic societies. We previously demonstrated the beneficial effects of a randomized prospective school-based health education program for low socioeconomic status Arab-Israeli kindergarten children.

**Methods:**

To examine whether the effects of our program on nutrition and physical activity knowledge and preferences, anthropometric measures, and fitness persisted one year after the end of intervention.

We were able to perform the one year follow-up in 203 kindergarten children (59% of our 342 original cohort; 85 control, 118 intervention).

**Results:**

At one year following the intervention BMI and BMI percentiles approached baseline level in both the intervention (16.4±0.2 kg/m^2^ and 61.5±2.4%, respectively) and control group participants (16.5±0.2 kg/m^2^ and 58.5±3.3%, respectively). Yet, a year after the end of the intervention, the decrease in BMI%ile from baseline was significantly greater in the intervention group (-7.8±1.5 vs. -1.9±1.9, p<0.012). Nutritional and physical activity knowledge and preferences, and physical fitness remained significantly elevated in the intervention compared to the control group participants.

**Conclusions:**

The beneficial effects of a kindergarten dietary-physical activity intervention applied by the kindergarten teachers, on nutrition and physical activity knowledge and preferences, fitness, and BMI percentile were evident one year after the end of intervention. This promising program may play a role in health promotion, prevention and treatment of childhood obesity.

## Background

Childhood obesity is increasing in westernized societies, with a higher prevalence in minorities and low socioeconomic status groups [1]. Childhood obesity in Israel is among the highest compared to European countries and the United States [2]. Relatively few studies examined the prevalence of childhood obesity in pre-school years. However, a similar trend of higher prevalence rate of kindergarten overweight and obesity among low socio-economic and minority communities was reported in the USA [3] and Israel [4,5]. The increase in the prevalence of preschool childhood obesity and the tendency of these children to become obese adults [6] suggests that health education interventions should start at young age.

The magnitude of the problem, organizational complexities and high costs make structured multi-disciplinary interventions possible only for a minority of the overweight children, therefore, the need for school-based interventions, were children spend a significant amount of their time, is warranted. Surprisingly, relatively few childhood obesity school-based preventive and therapeutic programs were reported [7], especially in the elementary and preschool children, showing a promising 10% reduction in the number of overweight children [8]. However, the long-term effect of these interventions is not known. Accordingly, a recent Cochrane review emphasized the lack of pre-school childhood obesity preventive and therapeutic interventions [9].

Israel is a diverse country hosting many different religions. The largest minority in Israel is Arab-Israelis. The term Arab-Israeli refers to Muslim Arabs who were born in the state of Israel. Arab-Israelis consist 15.9% of Israel’s overall population, and 83% of the Arab population in Israel (additional 9% are Arab Christians, and 8% Arab-Druze).

Environmental, social, cultural, educational and genetic factors lead to a very high prevalence of obesity among this unique minority group (58% among males and 41.7% among females between ages 18–44, Israeli Ministry of Health publications 2009, Israel Central Bureau of Statistics). We recently reported the high prevalence of childhood overweight and obesity (29%), and the favorable effects of a combined nutritional-physical activity health education programs on nutritional and physical activity knowledge and preferences, physical fitness and BMI percentiles in low socioeconomic status, Arab-Israeli kindergarten children [10]. The aim of the present study was to examine the longer-term effects (one year) of this school-based health promotion intervention in the Arab-Israeli kindergarten children. We hypothesized that at least some of the interventional beneficial effects on nutrition and physical activity knowledge and preferences, reduced BMI percentiles, and improved fitness will be kept after one year.

## Methods

In Israel, Kindergartens are separated from the school system. However, we were able to reach a relatively high number of participants after one year because many of the participants continued to a neighboring elementary school. Out of the three hundred and forty-two healthy kindergarten children who participated in intervention program [10], we were able to perform the one year follow-up study in 203 kindergarten children (59%; 85 control, 118 intervention). Since usually, the transition from kindergarten to elementary school involves redistribution of the children to several schools, especially in low SES status area were people move often, being able to follow almost 60% of the initial cohort was satisfying.

The study was approved by the Institutional Review Board of the Meir Medical Center, and the Israeli Ministry of Education. Children were included after parental consent.

The study included graduates from 11 Kindergartens from low socioeconomic status Arab-Israeli communities in central Israel. The determination of socioeconomic status was determined using criteria set by the Israeli Central Bureau of Statistics [10]. We included in the study measurements of both control and intervention group participants that were performed at the beginning and at the end of the intervention and one year after the end of the intervention. No structured intervention was performed during the follow-up year. Measurements were performed by the same group of trained technicians that were blinded to the assignment of the participant to either the intervention or control group.

### Intervention design

The intervention protocol was designed by pediatricians, registered dietitians, an exercise physiologist, youth exercise coaches and the pre-school staff. The major challenge was to ensure the transferability of the intervention, so that if successful, it will be possible to apply it in a larger number of pre-schools from minority communities nationwide.

After randomization, the intervention group pre-school teachers attended an all day seminar in which they were acquainted with the program, and were trained by the study team so that all the nutritional aspects of the intervention and the majority of exercise classes were performed by the pre-school staff (i.e. teacher and assistant teacher). Teachers were given lectures, hands-on sessions (on nutrition and physical activity) and written material to familiarize them with the program and enable them to perform it in their pre-schools classes. During the intervention, kindergarten teachers were invited to two additional training days. The goal of these meetings was to collect feed-back on the program and to introduce new materials to the teachers. Adherence to the program was followed weekly by the study coordinator, a registered dietitian and by professional youth coach. Participants in the control group were informed that measurements are part of a survey on physical activity and nutrition of kindergarten children. Control group participants continued their regular kindergarten schedule.

Parents and children of the intervention groups only were invited for two “Health Festival” days that focused on the major themes of the program (introduction of healthy nutrition, prevention of childhood obesity and beneficial effects of exercise in children). The first festival was performed during the second month of the program, and the second festival was performed towards the middle of the program. The festivals included lectures given by the study team, and games in which the children and parents played together. At the end of school year intervention, teachers were invited to a summary meeting, in which they were given an update on the study results.

### Nutritional intervention

The nutritional intervention was designed mainly to improve nutritional knowledge and was based on the nutritional program “It Fits Me” (“Tafur Alay”) of the Israeli Ministry of Education (http://www.tafuralay.co.il). Briefly, the intervention consisted of topics like food groups, vitamins, healthy food choices, food preparation and cooking methods, and fast-food versus home cooking. The topics were taught through short lectures/talks, games and story reading. Topics, such as, what do popular Israeli foods contain? Fruits and vegetables, what is calcium and why it is important, special dietary consideration during holidays, and how to deal with food excess during celebrations, vacations, restaurants etc. were also covered. All topics were delivered by the preschool teachers and made appropriate to the cognitive and social development levels of kindergarten children. In addition, monthly flyers detailing nutritional information were sent home via the children. Children were asked to present the nutritional information to their parents, and parents were asked to discuss the information with their children.

### Physical activity program

All intervention children participated in a 45 minutes (divided to three 15 minute sessions) per day exercise training (six days/week). Once a week, the training was directed by a professional youth coach. During the rest of the week similar physical activity sessions were coordinated by the pre-school teacher and/or her assistant, as instructed during the seminars. The physical activity sessions were performed indoors and/or outdoors. The activities varied in duration and intensity, and were designed primarily as games to encourage enthusiasm and participation of the children. Endurance type activities accounted for most of the time spent in training [about 20% team sports (soccer, dodge ball), and 80% running games (tag, hide and seek, relays etc.)], with attention also given to coordination and flexibility skills. Children were encouraged by the study staff to increase their habitual after-school physical activity, and to reduce sedentary activities (e.g. television viewing, video games). Pre-school teachers were also given a CD collection of children songs, written by a famous Israeli children song writer, related to the topic of nutrition and exercise.

### Anthropometric measurements

Standard, calibrated scales and stadiometers (Seca 769, Hamburg, Germany) were used to determine height, weight, and body mass index (BMI-kg/m^2^). Participants were asked to void and remove shoes and heavy clothing before measurement. Children were measured twice and the average was recorded for analysis. A third measurement was performed when a difference of >0.1 kg or 0.5 cm was detected, and the two closest measurements were averaged. Measurements were performed by an experienced technician who was blinded to the group assignment. Since BMI changes with age and due to the length of the intervention (one school year and additional one year follow-up), BMI-for-age percentile was calculated according to the Center for Disease Control growth charts [11].

### Nutritional and physical activity knowledge and preferences evaluation

Nutritional and physical activity knowledge and preferences were evaluated by a photo-pair food and exercise -pairing questionnaire developed by Calfas et al. [12]. The questionnaires were completed individually, and the assessors were blinded to the assignment of the children. The same assessor performed the pre, post and one year follow-up evaluations in each participant.

At the beginning of the test each child was asked if he/she knew what healthy means, and was provided with an age appropriate explanation, such as, “Being healthy means that you can play outside, you do not get sick, and you feel good”. To determine knowledge, children were asked to choose a doll which they were supposed to help stay healthy. The doll was used so that the child would assume a more caretaking approach and to make it less likely that they make choices purely based on personal preferences. Then children were presented with 15 photo-pairs pictures. In each set of pictures, presented on a laptop computer, one picture signified a healthy choice and the other an unhealthy one. Children were asked to identify which food/activity of the pair would “make the doll healthy and grow big and strong”.

To determine preferences children were presented with the same 15 pairs of pictures and were asked to point to food/activity they best like. This visual instrument was found previously appropriate for this age group and was validated on a similar group of children in the United States [12]. The order of knowledge or preferences testing was randomly assigned using computerized program. A score (0–100%) for nutrition and for physical activity knowledge and preferences was calculated and used for statistical analysis.

### Fitness assessment

Fitness was assessed using the shuttle run test. The test usually consists of shuttle running at increasing speeds between two markers placed 20 m apart. A portable compact disc (Sony CFD-V7) dictates the test pace by emitting tones at appropriate intervals. Due to the participants’ young age, and since we were not allowed to test the children outside the kindergarten, we modified the test and used the same pacer, but reduced the running distance to 10 m. Each participant was required to be at one of the ends of the 10 m course at the signal. A start speed of 8.5 km/hour was maintained for one minute, and thereafter the speed was increased 0.5 km/hour every minute. The same protocol was used at the one year follow-up test. The test score achieved was the number of laps completed before the subject either withdrew voluntarily from the test or failed to arrive within a meter of the end line on two consecutive tones. All children were encouraged throughout the test by the staff to achieve their best performance.

### Statistical analysis

Two sample t-test was used to determine baseline differences between the control and intervention groups. A two-way repeated measure ANOVA was used to compare effects of the intervention on body weight, height, BMI, BMI percentiles, nutrition and physical activity knowledge and preferences and fitness between the intervention and control participants with time serving as the within group, and intervention as the between group factor. When differences between the two groups were identified, a mixed model analysis was performed, to ensure no class effect. Statistical significance was taken at p<0.05. Data are presented as mean ± standard error of the mean (SEM).

## Results

Subject characteristics are summarized in Table 1. No significant differences in age, gender, BMI, nutritional and physical activity knowledge and preferences were found between groups prior to the health promotion intervention (Table 1). Unlike our original baseline cohort, BMI percentile was higher and fitness was lower in the control group that was available for follow up. There was no difference in the relative number of overweight and obese children in the two groups.

**Table 1:** Anthropometric and fitness characteristics of the study participants at baseline

	**Control (n=85)**	**Intervention (n=118)**
Age (years)	5.5±0.03	5.4±0.03
Gender (M/F,%)	55/45	58/42
Weight (kg)	19.8±0.3	19.6±0.2
Height (cm)	110.8±0.5	109.4±0.4
BMI (kg/m^2^)	16.1±0.2	16.4±0.1
BMI Percentile	61.1±3.1	69.4±2.0*
Overweight BMI%ile 85–95	18/85 21.2%	23/118 19.5%
Obese BMI%ile > 95	7/85 8.2%	10/118 8.5%
**Overweight and obese** BMI%ile > 85	25/85 30.0%	32/118 28.0%
Fitness (# laps)	27±1	35 ±1*

* p<0.05.

### One year intervention

Following the one year intervention there was a significant decrease (p< 0.0001) in BMI in both the intervention (from 16.4±0.1 to 15.7±0.1 kg/m^2^) and control participants (from 16.1±0.2 to 15.8±0.2 kg/m^2^), with a significant between group change difference (control; -0.33±00.8, intervention; -0.73±0.06, p=0.005). There was also a significant decrease (p< 0.0001) in BMI percentile in both the intervention (from 69.4±2.0 to 53.4±2.5%) and control participants (from 61.4±3.1 to 53.5±3.7%), with a significant between group difference in BMI%ile change (control; -8.6±1.9%, intervention; -16.5±1.4%, p=0.001).

**Figure 1:**
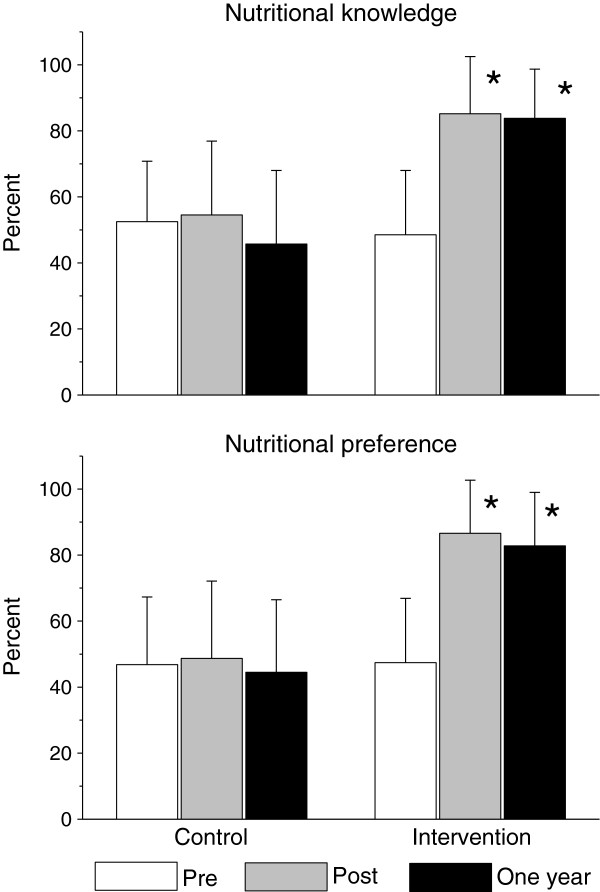
Long-term effects of the health promotion intervention on nutritional knowledge and preferences.

**Figure 2:**
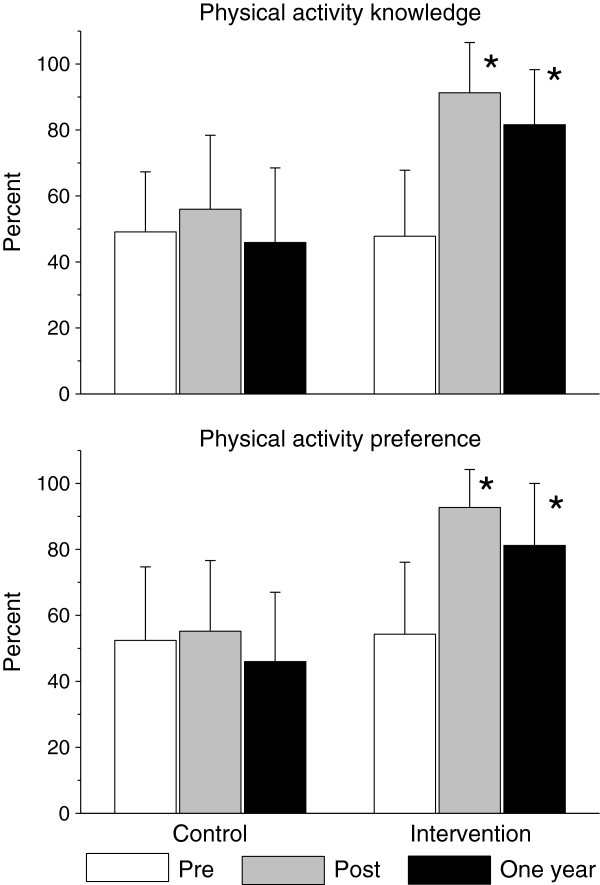
Long-term effects of the health promotion intervention on physical activity knowledge and preferences.

**Figure 3:**
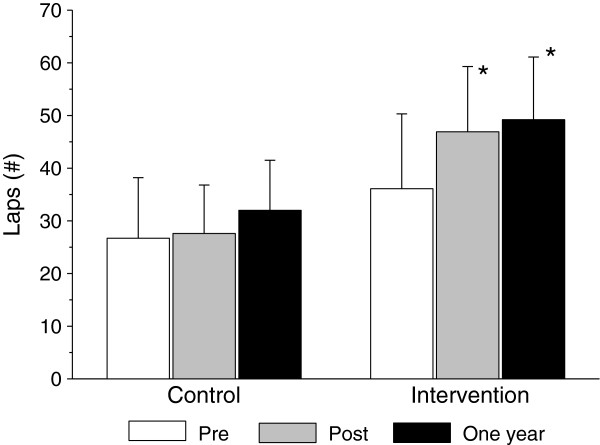
Long-term effects of the health promotion intervention on physical fitness.

Changes in nutritional knowledge and preferences are summarized in Figure 1. There was a significant increase in nutritional knowledge and preferences in the intervention compared to control group participants. Changes in physical activity knowledge and preferences are summarized in Figure 2. There was a significant increase in physical activity knowledge and preferences in the intervention compared to control group participants. Physical fitness improved significantly greater in the intervention compared to the control group participants (Figure 3).

### Long-term (one year) effects

**Figure 4:**
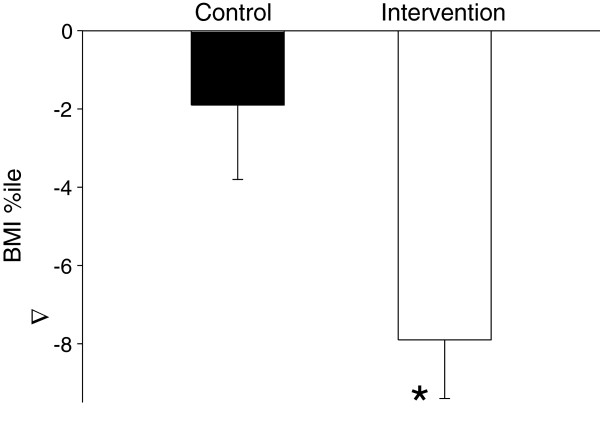
BMI percentile changes from baseline in the intervention and control, one year following the completion of the intervention.

One year following the health promotion intervention BMI and BMI percentiles approached baseline level in both the intervention (16.4±0.2 kg/m^2^ and 61.5±2.4%, respectively) and control group participants (16.5±0.2 kg/m^2^ and 58.5±3.3%, respectively). Yet, a year after the end of the intervention, the decrease in BMI%ile from baseline was significantly higher in the intervention group (Figure 4). Nutritional (Figure 1) and physical activity (Figure 2) knowledge and preferences, and physical fitness (Figure 3) remained significantly elevated in the intervention compared to the control group participants.

## Discussion

We recently demonstrated that a school-based combined nutritional and physical activity intervention in Arab-Israeli kindergarten children was associated with an increase in nutritional and physical activity knowledge and preferences, improved fitness and greater decrease in BMI percentiles [10], emphasizing that the school system might be a good platform to reach children in need and implement programs to treat and prevent childhood obesity in minority populations. In the present study, we aimed to determine the longer-term (one year) effects of this program in Arab-Israeli kindergarten children. Encouragingly, our findings indicate that the beneficial effects on nutrition and physical activity knowledge and preferences and on BMI, BMI percentile and physical fitness persist one year after the end of the intervention.

One of the major findings of the present study was the striking intervention-induced and the long-term improvement in nutrition and physical activity knowledge and preferences. Notably, the baseline nutrition and physical activity knowledge of the Arab-Israeli kindergarten children was distinctly lower compared to previously reported scores of Jewish kindergarten children from middle-high socio-economic communities [13], but similar to Jewish kindergarten children from low socio-economic communities [5]. This highlights the large gap in health related knowledge between minority and low socio-economic children compared to high socio-economic children in as early as the kindergarten years, representing, probably, the large environmental contribution to the general knowledge of children in this age. This suggests that increasing nutritional and physical activity knowledge should be one of the first steps in any childhood obesity intervention for this unique population. Increased knowledge may lead to improved preferences and to healthier nutritional life-style and increased physical activity. Encouragingly, post intervention, and even more importantly, one year following the intervention, nutrition and physical activity knowledge and preference scores in the intervention group participants were similar and even higher compared to Jewish high socio-economic class kindergarten children [13].

The increased sedentary lifestyle in western societies is a major contributor to the higher prevalence of childhood obesity [14,15]. Therefore, physical activity was a main focus of the intervention. This resulted in a significant increase in fitness among the intervention group participants. Importantly, physical fitness was improved in both genders and in the normal weight, overweight and obese Arab-Israeli kindergarten children [10]. This is consistent with our previous findings following a similar intervention in both low [5] and moderate-high socio-economic Jewish kindergarten children [4] from the same geographic region. More importantly, the present study indicates that physical fitness remained significantly elevated one year after the end of the intervention. We previously demonstrated [4] in high socio-economic kindergarten children that a similar intervention led to a significant increase in physical activity during both school and after-school hours, suggesting that the importance of habitual physical activity was incorporated. Physical activity level of the control and intervention group participants during the year after the intervention was not monitored; however, the present study results clearly show that physical fitness remained improved in the intervention group participants. The improved fitness both immediately and one year after the intervention may have both psychosocial and other health related effects, such as improved lipid profile, insulin sensitivity, decreased blood pressure and reduced coronary heart disease risk later in life [16]. These were not determined in the present study.

Ages 4–7 are characterized by a natural decrease followed by an increase in BMI percentiles [17]. Thus, the decrease and later increase in BMI percentiles of the control group participants was not surprising. One year following the intervention, the increase in BMI percentiles of the intervention group participants did not reach pre-intervention levels, while BMI percentiles of the control group participants returned to baseline level, and this difference was statistically significant. This suggests that nutrition and physical activity knowledge and preferences and improved fitness, translate to a long-term significantly greater reduction in BMI percentiles.

Adult studies indicate that the long-term effects of weight reduction programs have been very disappointing [18]. We previously demonstrated [19] that the favorable short term effects of a combined hospital based, structured dietary-behavioral-physical activity intervention on BMI, age-adjusted BMI percentile, body fat, habitual activity and fitness level remained significant one year after the intervention in obese children. The present study indicates that similar one year follow-up outcome was found following a school-based program.

Uniquely, the school-based combined nutritional-physical activity intervention was incorporated into the existing pre-school core curriculum and delivered mainly by the pre-school staff, and not by external professional personnel. Therefore, the beneficial short and long-term effects of the program on nutrition and physical activity knowledge and preferences, BMI percentiles and fitness in the minority community of Arab-Israeli children did not require major time or financial investment. We, therefore, believe that this program may serve as a model in the nationwide campaign against sedentary lifestyle and inactivity in kindergarten and pre-schools even in minority populations. Since we believe that the kindergarten staff are key players in the success of such a program, their attitude and long-term adherence toward the intervention should be further studied.

## Conclusions

Although the one year follow-up results are promising, the longer term effects (e.g. 3, 5 or 10 years) of this intervention are yet to be determined. Encouragingly, however, weight reduction multi-disciplinary pediatric interventions seem more successful than adult programs in maintaining the beneficial effects for 5 or 10 years [20,21]. Moreover, since nutrition and physical activity were not monitored during the one year follow-up after the end of the intervention, the relative contribution of each factor to the success of the intervention cannot be determined.

## Abbreviations

BMI: Body mass index

## Competing interests

Authors state no competing interests.

## Authors’ contributions

DN and AE conceived of the study, participated in its design and coordination and drafted the manuscript. DG, MP and NI made substantial contributions to or acquisition of data, and revised the manuscript. YM made substantial contributions to the analysis and interpretation of data, and assisted in drafting the manuscript. All authors read and approved the final manuscript.

## Pre-publication history

The pre-publication history for this paper can be accessed here:

http://www.biomedcentral.com/1471-2431/13/45/prepub
